# Community Level Physiological Profiles (CLPP), Characterization and Microbial Activity of Soil Amended with Dairy Sewage Sludge

**DOI:** 10.3390/s120303253

**Published:** 2012-03-07

**Authors:** Magdalena Frąc, Karolina Oszust, Jerzy Lipiec

**Affiliations:** Institute of Agrophysics, Polish Academy of Sciences, Doświadczalna 4, 20-290 Lublin 27, Poland; E-Mails: k.oszust@ipan.lublin.pl (K.O.); j.lipiec@ipan.lublin.pl (J.L.)

**Keywords:** microbial diversity, community level physiological profiles, Biolog EcoPlate, dairy sewage sludge, soil

## Abstract

The aim of the present work was to assess the influence of organic amendment applications compared to mineral fertilization on soil microbial activity and functional diversity. The field experiment was set up on a soil classified as an *Eutric Cambisol* developed from loess (South-East Poland). Two doses of both dairy sewage sludge (20 Mg·ha^−1^ and 26 Mg·ha^−1^) and of mineral fertilizers containing the same amount of nutrients were applied. The same soil without any amendment was used as a control. The soil under undisturbed native vegetation was also included in the study as a representative background sample. The functional diversity (catabolic potential) was assessed using such indices as Average Well Color Development (AWCD), Richness (R) and Shannon–Weaver index (H). These indices were calculated, following the community level physiological profiling (CLPP) using Biolog Eco Plates. Soil dehydrogenase and respiratory activity were also evaluated. The indices were sensitive enough to reveal changes in community level physiological profiles due to treatment effects. It was shown that dairy sewage amended soil was characterized by greater AWCD, R, H and dehydrogenase and respiratory activity as compared to control or mineral fertilized soil. Analysis of variance (ANOVA) and principal component analysis (PCA) were used to depict the differences of the soil bacterial functional diversity between the treatments.

## 1. Introduction

Increasing levels of urbanization and industrialization have a significant effect on the amount of sewage produced worldwide. That is also reflected in the upswing of the number of sewage treatment plants that produce continually increasing amounts of sewage sludge. This vast volume of sewage sludge must be neutralized and utilized in an appropriate manner [1,2]. Sewage sludge application to the soil is considered to be beneficial due to the productivity of ecosystems through the introduction of nutrients (N, P, K) and the soil structure by the addition of organic matter [3].

Land application of sewage sludge is one of the most important disposal alternatives, but potential availability of heavy metals, toxic compounds and pathogenic bacteria often restricts its uses [4–6]. Some studies showed that the sludge is low in heavy metals, toxic organic compounds and pathogenic bacteria, viruses, parasites and can be used in agriculture without increasing environmental pollution [5,7]. Sludge amended soil has different physico-chemical and biological properties.

Monitoring the soil microbial community and activity can be a powerful tool for understanding basic and applied ecological contexts. Rapid growth of microorganisms in the soil allows for comprehensive study of community interactions much more readily than in a plant or animal system [8], and, essentially foster insight into experimental manipulations and successional processes within reasonable time frames. Enzymes released by microorganisms during the degradation of sewage sludge play a key role in the biological and biochemical transformations that take place in the soil environment [8]. As reported by several authors [7,9–11], the biological parameters of soil are largely modified by environmental or anthropogenic factors, and may be a potential indicator of ecological stress [12,13]. Microbial enzymes are also responsible for the decomposition of complex organic compounds [14].

Nevertheless, it seems necessary to take under consideration the rapid community-level cultural approach, subsequently called CLPP, developed by Garland and Millis [15]; for 95 different carbon sources (GN plates). The study of Haack *et al*. [16] and Lehman *et al*. [17] indicated that fewer than the 95 substrates is sufficient to evaluate changes in functional microbial community in terrestrial ecosystems. Following this approach, Insam [18] proposed a 96-well microplate with 31 substrates plus control, each in three replications (EcoPlate) as a new set of substrates for community characterization in environmental samples. The CLPP method provides an exciting opportunity to overcome the drawbacks of alternative time consuming culture-based analyses or biochemical tests [19,20]. The CLPP approach is frequently employed to determine the effect of various environmental factors on the biological status of particular soil sites by following catabolic traits [16]. On the other hand as Garland [8,21] noticed, the metabolic growth response, which involves cooperative as well as competitive effects in Biolog EcoPlates wells, might be a major drawback of the CLPP method.

It is assumed, that agricultural management practices must be compatible with the principle of sustainable development. They must also promote long-term fertility and productivity providing nutrient supplements, fixed and mineralized by the microbiota [22,23]. Organic amendments act as energy sources [24], which change microbial biodiversity and activity [25–27]. The estimation of microbial functional diversity by using CLPP has been reported as a sensitive approach to detect modifications due to soil management [26,28]. The Biolog EcoPlates™ system allows testing for a number of ecologically relevant substrates with replication [26].

The aim of the present work was to assess the response of soil microbial activity and functional diversity to dairy sewage sludge application and mineral fertilization. The study included determination of dehydrogenase and respiratory activity and CLPP using the Biolog EcoPlates™ system. This research offers important data that supports the application of sewage sludge to agricultural land.

## 2. Experimental Section

### 2.1. Site Selection and Description of the Experiment

The field experiment was conducted on an *Eutric Cambisol* soil type developed from loess. The experiment sites were situated in the vicinity of a dairy sewage treatment plant in Krasnystaw, South-East Poland (50°59′4″N, 23°8′5″E). The experiment was established in an agricultural field that had been in conventional tillage agriculture for at least past 50 years with crop rotations including selected cereals, root crops and papilionaceous crops. The research area has rather uniform soils with respect to genesis and textural composition [2]. Some chemical and texture characteristics of the soil are given in Table 1.

The treatments were as follows:

- Undisturbed native vegetation established on the experimental plots abandoned from agricultural use in 1996 (UND),
- Soil with neither, mineral fertilization or dairy sewage sludge, control (C),
- Mineral fertilization amendment 130 kg·N·ha^−1^, 80 kg·P·ha^−1^, 120 kg·K·ha^−1^ (termed MFSD–mineral fertilization low dose),
- Mineral fertilization amendment 170 kg·N·ha^−1^, 100 kg·P·ha^−1^, 160 kg·K·ha^−1^ (termed MFLD–mineral fertilization large dose),
- Dairy sewage sludge amendment 20 Mg·ha^−1^ plus 80 kg·K·ha^−1^ in order to complement the amount of nutrients to the same level as in MFSD (termed DSSSD–dairy sewage sludge small dose),
- Dairy sewage sludge, 26 Mg·ha^−1^ plus 110 kg·K·ha^−1^ in order to complement the amount of nutrients to the same level as in MFLD (termed DSSLD–dairy sewage sludge large dose).

The main characteristics of the dairy sewage sludge (DSS) are given in Table 2. The DSS was applied in autumn 2010 and plowed to a depth of 30 cm. A completely randomized block with four replicates was used as the experimental design. Experimental plots were sown with winter wheat in 2010. Soil samples for analyses were taken from the depth of 0 to 20 cm one year after application of amendments after winter wheat harvest. The soil samples were screened through sieves with 2 mm mesh and then used for EcoPlates (Biolog, Hayward, CA, USA) and microbial activity analyses.

### 2.2. Chemical Analysis

Soil total organic carbon (C_org._) was determined using the Tiurin method [31]. pH was measured by the electrometric method from a soil aqueous extract. Total N was measured by the Kjeldahl method and total P by flow spectrophotometric method (SKALAR San System) and total K by flame emission spectrometry method (AAS-3 CarlZeiss Jena) after wet sulphuric acid digestion of samples. The heavy metals were extracted with aqua regia and measured with atomic absorption spectroscopy (AAS). The same methods were used with respect to soil and dairy sewage sludge.

### 2.3. Microbial Activity

Dehydrogenase activity was determined according to Thalmann [32], using TTC (triphenyl tetrazolium chloride) as a substrate, in Tris-HCl buffer (pH 7.4). Incubation was conducted for 96 h at 30 °C. Enzymatic activity was determined spectrophotometrically at 485 nm, with reference to methanol. The respiratory activity was determined by substrate-induced respiratory according to Rühling and Tyler [33], 10 mg glucose g^−1^ dry soil was added to obtain maximum initial respiratory response.

### 2.4. Community Level Physiological Profiling (CLPP) Analysis

Biolog EcoPlates, that are 96-well plates, containing 31 different carbon sources plus a control well, in three replications. Tetrazolium violet redox dye was used for each well as a color indicator if added microorganisms utilize the substrates [18,34]. One g portions of soil were shaken in 99 mL of distilled sterile water for 20 min at 20 °C and then were incubated at 4 °C for 30 min [35]. Next 150 μL of each sample were inoculated into each well of Biolog EcoPlates and incubated at 25 °C. The rate of utilization was indicated by the reduction of the tetrazolium, a redox indicator dye that changes from colorless into purple [17,25]. Data were recorded with a plate reader at 590 nm every 24 h until 120 h.

Microbial response in each microplate that expressed average well-color development (AWCD) was determined as follows [22]:

$$\text{AWCD}=\sum {\text{OD}}_{i}/31$$

where OD_i_ is optical density value from each well, corrected subtracting the blank well (inoculated, but without a carbon source) values from each plate well [15,36].

Richness (R) values were calculated as the number of oxidized C substrates, and the Shannon–Weaver index values (H) (*i.e.*, the richness and evenness of response) were calculated using an OD of 0.25 as threshold for positive response [8]. The Shannon–Weaver index was calculated as follows:

$$H=-\sum p_{i}({\text{lnp}}_{i})$$

where p_i_ is the ratio of the activity on each substrate (OD_i_) to the sum of activities on all substrates ∑OD_i_. Plate readings at 48 h of incubation were used to calculate AWCD, R and H, since it was the shortest incubation time that allowed the best resolution among the treatments.

We used the following five groups of carbon substrates: (1) carbohydrates; (2) carboxylic and acetic acids; (3) amino acids; (4) polymers; and (5) amines and amides according to Weber and Legge [37] (Table 3). In our study carbon sources originally grouped as miscellaneous by Zak *et al*. [38], were included in the carbohydrates category according to Weber and Legge [37]. For each series the corrected absorbance values of the substrates were summarized and expressed as a percentage of total absorbance value of the plate corresponding to a particular treatment [37,39].

### 2.5. Statistical Analysis

Average well-color development, richness and Shannon–Weaver indices, dehydrogenase and respiratory activity were analyzed by ANOVA and mean comparisons between treatments were performed using Tukey’s mean separation test at P < 0.05. Principal component analysis (PCA) to analyze CLPPs was performed on normalized and transformed absorbance data for each well, according to Weber *et al*. [36]. Transformed data were used for PC analysis because of two fundamental properties of the data set: normality and homoscedasticity were not met. Normality of the data was evaluated though formal statistical Shapiro-Wilk test. However, homoscedasticity, that is homogeneity of variance was performed by Hartley test. All statistical analyses were performed with Statistica 9.0 software (2010).

## 3. Results and Discussion

**Figure 1.**
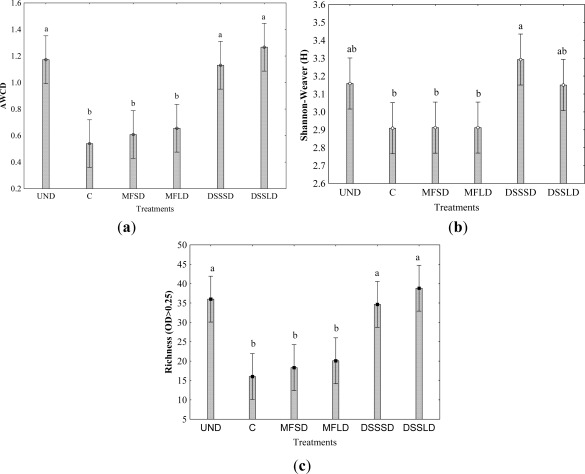
(**a**) Average well-color development (AWCD); (**b**) Richness (R) and (**c**) Shannon–Weaver index (H) of metabolized substrates in Biolog EcoPlate™ after amendment application. Explanations: UND–undisturbed native vegetation, C–control soil without mineral and organic fertilization, MFSD–mineral fertilization amendment, small dose, MFLD–mineral fertilization amendment, large dose, DSSSD–dairy sewage sludge, small dose, DSSLD–dairy sewage sludge, large dose, n = 3. Vertical bars represent the standard error of the mean. Treatment means separated by different letters are significantly different (Tukey’s mean separation test, P < 0.05).

As demonstrated by Figure 1, the AWCD, R, H indexes from the dairy sewage sludge amended soil were significantly (P < 0.05) greater than in the mineral fertilized and control soil. This indicates a greater rate of substrate utilization (catabolic potential) by the microbial community and greater functional diversity in the sewage-amended than in mineral fertilized soils. However, there were no significant differences between the indices of dairy sewage sludge (DSS) amended soil and soil under long-term undisturbed native vegetation. The results indicated higher values of AWCD, H and R indices in undisturbed native soil compared to the control. These differences were probably due to the presence of more complex organic material such as plant and root debris in undisturbed site and also root exudates of a greater variety of plants species growing on undisturbed native than control soil, on which only monoculture of winter wheat was cropped.

Baudoin *et al*. [40] indicated that root exudates stimulated bacterial proliferation and increased microbial activity and communities. At the undisturbed site a variety of different plant species and residues may result in organic compounds, which may increase potential metabolic diversity.

In order to determine the extent of differentiation of treatments from the control and the undisturbed sites with regard to carbon source catabolism, each of the amendment application rates was subjected to principal component analysis. The first and the second principal component (PC1 and PC2) explained 36.7% and 26.5% of data variance, respectively, with a small dose of the mineral and organic amendments. At the large dose of the amendments the corresponding values were 27.6% and 20.3% for PC1 and PC2 (Table 4). The plots amended with dairy sewage sludge and the undisturbed native vegetation plots clustered together, when large dose of the amendments was considered. Soil amended with dairy sewage sludge was more similar to the undisturbed site and different from plots amended with mineral fertilizers and the control. This indicates differences in microbial communities between the two groups of treatments of greater and lower sludge application, respectively. The carbon sources significantly correlated with PC1 (R > 0.7) at small and large doses of amendments are shown in Table 4. These were responsible for the differences among samples. Few of the carbohydrates showed a strong negative correlation with PC1 for the soil treated with small dose of amendments. In contrast, strong positive correlations were observed between amino acids, carboxylic and acetic acids and PC1. For large dose of amendments carbohydrates showed a strong positive correlation with PC1. Some negative correlations were also observed between few carboxylic and acetic acids, amino acids and amines and amides and PC1. However, no specific correlations were seen for the other carbon source groups.

**Figure 2.**
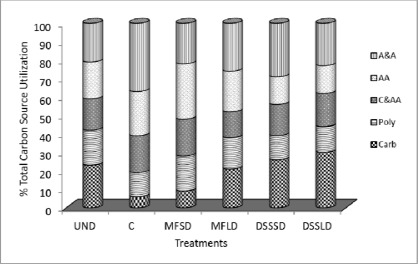
Percent of total carbon source utilization response tracked due to treatment type, for the different guilds–amines and amides (A&A), amino acids (AA), carboxylic and acetic acids (C&AA), polymers (Poly) and carbohydrates (Carb). Explanations: UND–undisturbed native vegetation, C–control soil without mineral and organic fertilization, MFSD–mineral fertilization amendment, small dose, MFLD–mineral fertilization amendment, large dose, DSSSD–dairy sewage sludge, small dose, DSSLD–dairy sewage sludge, large dose.

The substrates situated on microtiter plates were divided into five main groups: carbohydrates, carboxylic and acetic acids, amino acids, polymers, amines and amides. Their relative contributions in soil amended with dairy sewage sludge and mineral fertilization were examined. Figure 2 presents the percentage of total carbon source utilization for each guild (see Table 3 for class groupings) after amendment application.

Comparing the guild utilizations for particular treatments the group shifts are similar for the three treatments (DSSSD, DSSLD and UND). These results suggest that bacterial communities in all treatments are similar. The shifts of dairy sewage sludge amended and undisturbed soil are different from the control and mineral fertilized treatments shifts, which clearly confirms a microbial response to the perturbation of treatments. The data suggest that composition of microbial communities shows susceptibility to change under studied treatments. Dairy sewage sludge application to the soil seems to boost its microbial communities and increase its predisposition to recover it towards UND communities. We found a strong contribution of carbohydrates and amino acids throughout the DSS amended and undisturbed soils. The average absorption value of carbohydrates declined within increase in the average absorption value of amino acids in MFSD and amines and amides in control soil. It suggests that carbohydrates and amino acids were incorporated into soil with dairy sewage sludge. The results indicated that the carbohydrates (Carb) and amines and amides (A&A) groups were the most intensively metabolized, especially in the sewage sludge-treated soil and control soil, respectively. Generally, the soil under undisturbed native vegetation showed similar utilization level of all the groups of substrates. The results also indicated that dairy sewage sludge amendment appreciably increased the level of carbohydrates and decreased the level of amines and amides usage compared to control soil. There was no significant effect of the amendments dose on substrate categorized utilization pattern.

**Figure 3.**
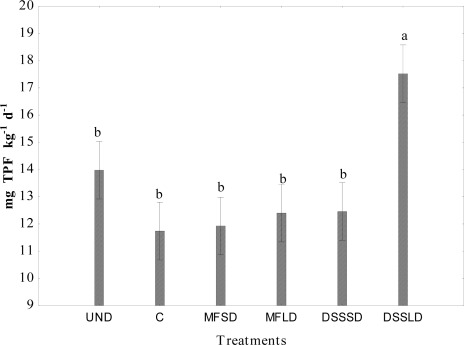
Dehydrogenase activity in soil 1 year after amendment application. Explanations: UND–undisturbed native vegetation, C–control soil without mineral and organic fertilization, MFSD–mineral fertilization amendment, small dose, MFLD–mineral fertilization amendment, large dose, DSSSD–dairy sewage sludge, small dose, DSSLD–dairy sewage sludge, large dose, n = 3. Vertical error bars represent the standard error of the mean.

**Figure 4.**
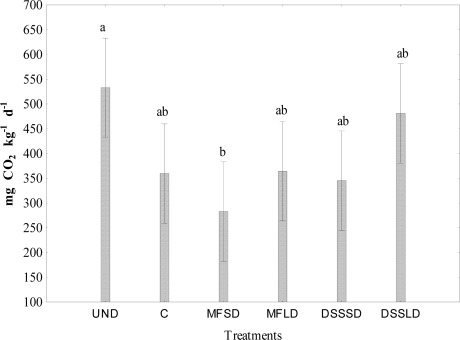
Respiratory activity in soil 1 year after amendment application. Explanations: UND–undisturbed native vegetation, C–control soil without mineral and organic fertilization, MFSD–mineral fertilization amendment, small dose, MFLD–mineral fertilization amendment, large dose, DSSSD–dairy sewage sludge, small dose, DSSLD–dairy sewage sludge, large dose, n = 3. Vertical error bars represent the standard error of the mean.

Low carbon availability is frequently a limiting factor for microbial growth [30,41,42]. It is worth noting that the use of the Biolog Eco-Plate™ assay with carbon substrates in our study showed changes in the microbial functional diversity after one year following the dairy sewage sludge application. These results are consistent with findings from an earlier experiment (Gomez *et al*. [24]), were significant increases in AWCD, R and H were measured several months after organic amendment application. Moreover, we noticed that the above mentioned CLPP indices were highly positively correlated to each other (r = 0.74–0.99) (Table 5). Comparison of Figures 1, 3 and 4 indicates that treatment effects on microbial activity were reflected similarly by CLPP indices, dehydrogenase activity and respiratory activity. Dehydrogenase activity under DSSLD with large dose of the sewage sludge was significantly greater (p < 0.05) compared to other treatments. Positive effect of dairy sludge application on dehydrogenase activity was also observed in our earlier field study [43]. As can be seen from Table 5 the dehydrogenase activity was significantly correlated with AWCD (p < 0.01).

Community level physiological profiles showed that potential metabolic diversity was significantly enhanced in the soil treated with dairy sewage sludge compared to chemical fertilizer as shown by values of AWCD and R index. Microbial activity, expressed as AWCD, was higher in dairy sewage sludge treated soil and undisturbed treatment compared to the control and mineral fertilized soils. Taking into consideration that the amount of nutrients introduced with the dairy sewage sludge and mineral fertilizers was the same, the results indicate that the increased functional diversity may be due to an additional supply of carbon to soil through the sludge on the DSS plots.

CLPP analysis using principal component analysis showed that the DSSLD and the undisturbed soils were different than the control and MFLD soils by PC1 (the ordinate principal component analysis plot), explaining 36% of the variance. This implies differences in microbial communities between the two groups of treatments that can result from a greater availability of C in the former. Also, PCA performed on data from each of the treatments with small dose applications showed that plots amended with dairy sewage sludge and soil under undisturbed native vegetation grouped together and differentiated from the control and mineral fertilized soil. This suggests that greater carbon availability due to dairy sewage sludge incorporation could explain the increase in microbial functional potential. Second principal component analysis was correlated with three carbon sources (α-ketobutyric acid, d-malic acid and phenylethylamine) for small dose of amendments and two carbon sources (l-phenylalanine, putrescine) for large dose of amendments from the 30 studied. These results indicate that carbon sources like carboxylic acids and amines had influence on community level physiological profiles, suggesting grater carbon availability from these sources.

Using the Biolog EcoPlate™ assay with carbon substrates allowed detection of the changes in the microbial functional diversity after dairy sewage sludge application. The results are in agreement with findings of a study of Gomez *et al*. [24] evaluating the soil microbial functional diversity in response to vermicompost and various types of manures as amendments. Also Mechri *et al*. [44] and Suhadolc *et al*. [45] investigated the short-term effect of organic wastes on the soil microbial communities. They indicated that agronomic application of organic waste and sewage sludge can cause the changes in microbial community structure and it may lead to changes in the patterns of C cycle in soil. The above changes promoted decomposition processes by influencing the physiology of soil microorganisms and increasing the catabolic profiles [46].

Organic amendment applications also affect soil chemical and physical properties [27,47,48]. However, microbial responses are considered to occur sooner due to the fact that soil biota is the labile fraction of soil organic matter engaged in the cycling of nutrients and energy [46]. Community level physiological profiles (CLPP) indicate the capacity of soils to utilize different carbon sources (carbohydrates, polymers, amino acids). The CLPP is a fast screening method to detect differences among treatments [34]. This method has been used widely to characterize microbial communities of different habitats, ranging from natural, native soil environments to organic, composts and sewage sludge amended soil [49,50]. In our study the greatest differentiation of carbon substrate utilization appeared in soil amended with the dairy sludge and in soil under native undisturbed vegetation. The substrates utilization pattern in this study showed that carbohydrate consumption increased the most in response to the application of the dairy sludge and to the associated alterations in the microbial activity and functions. Similar response was observed in earlier study of Bossio and Scow [51] after addition of straw to soil. Also the use of other carbon substrates present in the Biolog EcoPlate™ was greater from the sludge amendment plot than control or mineral fertilized plots. However, the highest utilization of all carbon substrates from the soil under native undisturbed vegetation indicates sustainability of the soil environment. This refers to the health of an agricultural land use system and the ability of this system to maintain its productive capability. The lowest differences in carbon utilization from all the substrates between soil under native undisturbed vegetation and the dairy sludge amended plots can be a result of enhanced microbial activity due to carbon supply through the sludge.

Following the Council Directive 86/278/EEC statement, sustainable practice should make use of the large amounts of organic matter and nutrients present in sludge, but at the same time should not affect the quality of soil. Therefore, for development of any sewage sludge management decisions it is important to identify its effects not only on soil chemical properties, but also on soil microbial activity and functions. The approach used in this study may help to meet the requirements when sewage sludge is applied in agriculture.

Overall the preliminary results indicate that the use of sewage sludge containing organic matter and valuable nutrients can be a useful opportunity for its sustainable management for improvement of microbial community and soil fertility. Moreover, applicability of the sludge used in this study can be enhanced by its low concentrations of heavy metals (Table 1) that are below the limits established by the European Union for land application [11,29,30].

## 4. Conclusions

The results of this study showed the following:

1. The indexes AWCD, R and H derived from the Biolog EcoPlates System are sensitive indicators of the impact of the dairy sewage sludge application and mineral fertilization on soil microbial activity.
2. Addition of the dairy sewage sludge to the soil promotes the functional diversity (catabolic potential) of the soil microbial population and at the same time can be a useful opportunity for its recycling. Applicability of the sludge used in this study can be enhanced by its low concentrations of heavy metals.
3. The close correlation between catabolic potential of microorganisms and functional microbial diversity was observed in soil amended with dairy sewage sludge and mineral fertilization.
4. Continuation of the experiments will allow determining how the dairy sewage sludge may affect microbial community and functional potential in a longer time span.

## Figures and Tables

**Table 1.** Characteristics of the soil used in the experiment.

**Parameter**	EU Limit Values* (mg·kg^−1^)	**Value**
pH	-	6.4
C_org._ (g·kg^−1^dwt)	-	13.5
N_tot._ (g·kg^−1^) dwt	-	1.6
C:N	-	8.3
P_tot._ (g·kg^−1^ dwt)	-	18.3
K_tot._ (g·kg^−1^ dwt)	-	26.8
Zn (mg·kg^−1^ dwt)	150–300	28.7
Cd (mg·kg^−1^ dwt)	1–3	0.16
Cu (mg·kg^−1^ dwt)	50–140	7.16
Pb (mg·kg^−1^ dwt)	50–300	10.3
Ni (mg·kg^−1^ dwt)	30–75	10.1
Cr (mg·kg^−1^ dwt)	-	18.4
Hg (mg·kg^−1^ dwt)	-	0.09
Sand (g·kg^−1^)	-	80
Silt (g·kg^−1^)	-	470
Clay (g·kg^−1^)	-	450

* EU limit values for heavy metal when used as agricultural soils [29,30].

**Table 2.** Characteristics of the dairy sewage sludge used in the experiment.

**Parameter**	**Dairy Sewage Sludge**
pH	7.23
dry matter of sludge (g·kg^−1^)	121.3
C_org._ (g·kg^−1^dwt)	868.0
N_tot._ (g·kg^−1^ dwt)	54.4
P_tot._ (g·kg^−1^ dwt)	33.0
K_tot._ (g·kg^−1^ dwt)	15.6
Zn (mg·kg^−1^ dwt)	194.0
Cd (mg·kg^−1^ dwt)	0.0
Cu (mg·kg^−1^ dwt)	18.7
Pb (mg·kg^−1^ dwt)	5.3
Ni (mg·kg^−1^ dwt)	21.7
Cr (mg·kg^−1^ dwt)	14.1
Hg (mg·kg^−1^ dwt)	0.0

**Table 3.** Biolog Ecoplate™ carbon source guild groupings [37].

**Well No.**	**ID**	**C-source**	**Guild**
Well 1	C0	Water (blank)	
Well 2	C1	Pyruvic acid methyl ester	Carbohydrates
Well 3	C2	Tween 40	Polymers
Well 4	C3	Tween 80	Polymers
Well 5	C4	Alpha-cyclodextrin	Polymers
Well 6	C5	Glycogen	Polymers
Well 7	C6	D-cellobiose	Carbohydrates
Well 8	C7	Alpha-D-lactose	Carbohydrates
Well 9	C8	Beta-methyl-D-glucoside	Carbohydrates
Well 10	C9	D-xylose	Carbohydrates
Well 11	C10	i-erythritol	Carbohydrates
Well 12	C11	D-mannitol	Carbohydrates
Well 13	C12	N-acetyl-D-glucosamine	Carbohydrates
Well 14	C13	D-glucosaminic acid	Carboxylic & acetic acids
Well 15	C14	Glucose-1-phosphate	Carbohydrates
Well 16	C15	D,L-alpha-glycerol phosphate	Carbohydrates
Well 17	C16	D-galactonic acid-gamma-lactone	Carboxylic & acetic acids
Well 18	C17	D-galacturonic acid	Carboxylic & acetic acids
Well 19	C18	2-Hydroxy benzoic acid	Carboxylic & acetic acids
Well 20	C19	4-Hydroxy benzoic acid	Carboxylic & acetic acids
Well 21	C20	Gamma-hydroxybutyric acid	Carboxylic & acetic acids
Well 22	C21	Itaconic acid	Carboxylic & acetic acids
Well 23	C22	Alpha-ketobutyric acid	Carboxylic & acetic acids
Well 24	C23	D-malic acid	Carboxylic & acetic acids
Well 25	C24	L-arginine	Amino acids
Well 26	C25	L-asparagine	Amino acids
Well 27	C26	L-phenylalanine	Amino acids
Well 28	C27	L-serine	Amino acids
Well 29	C28	L-threonine	Amino acids
Well 30	C29	Glycyl-L-glutamic acid	Amino acids
Well 31	C30	Phenylethylamine	Amines/amides
Well 32	C31	Putrescine	Amines/amides

**Table 4.** Correlation of carbon sources with the first principal component (PC1) in soil treated with two different doses of amendments (R > 0.7).

**Amendment level**			
**SMALL DOSE**		**LARGE DOSE**	
	PC1 (36.7%)		PC1 (27.6%)
**Carbohydrates:**		**Carbohydrates:**	
Glucose-1-phosphate	−0.88	D-cellobiose	0.90
Alpha-D-lactose	−0.86	Beta-methyl-D-glucoside	0.84
Beta-methyl-D-glucoside	−0.84	Glucose-1-phosphate	0.70
D-cellobiose	−0.83		
D-xylose	−0.73	**Carboxylic & acetic acids:**	
N-acetyl-D-glucosamine	−0.71	Gamma-hydroxybutyric acid	0.77
		D-malic acid	−0.76
**Carboxylic & acetic acids:**			
D-galacturonic acid	0.96	**Amino acids:**	
D-galactonic acid-gamma-lactone	0.89	L-asparagine	−0.77
4-Hydroxy benzoic acid	0.78	L-phenylalanine	−0.72
**Amino acids:**		**Amines/amides:**	
L-serine	0.87	Putrescine	−0.75
L-asparagine	0.86		
L-arginine	0.84		

	PC2 (26.5 %)		PC2 (20.3%)
**Carbohydrates:**		**Carbohydrates:**	
i-Erythritol	0.84	Puruvic Acid Methyl Ester	0.88
**Carboxylic & acetic acids**		**Polymers**	
D-Malic Acid	−0.89	Tween 80	0.74
a-Ketobutiric Acid	−0.80		
**Amines/amides**			
Phenylethylamine	−0.87		

**Table 5.** Correlation coefficients (r) between examined microbial parameters.

	
	**AWCD**	**R**	**H**	**ADh**
**R**	0.74***			
**H**	0.99***	0.72***		
**ADh**	0.69**	N.S.	0.69**	
**RESP**	0.47*	N.S.	N.S.	N.S.

*, **, *** –indicated significance at the p > 0.05, p > 0.01, and p > 0.001 level, respectively; N.S.–no significant, AWCD index, R–richness index, H–Shanon-Weaver index, ADh–dehydrogenase activity, RESP–respiratory activity.
